# Comparison of intrathecal low-dose bupivacaine and morphine with intravenous patient control analgesia for postoperative analgesia for video-assisted thoracoscopic surgery

**DOI:** 10.1186/s12871-023-02350-3

**Published:** 2023-12-01

**Authors:** Miao Guo, Suhong Tang, Yixin Wang, Fengxia Liu, Lin Wang, Dawei Yang, Jianyou Zhang

**Affiliations:** 1 Department of Anesthesiology, Affiliated Hospital of Yangzhou University, Yangzhou, 225000 China; 2 Graduate School of Dalian Medical University, Dalian, 116000 China

**Keywords:** Intrathecal morphine, Postoperative pain, Thoracoscopy lobectomy, Pneumonectomy, Quality of recovery

## Abstract

**Background:**

Thoracoscopic surgical techniques continue to advance, yet the intensity of postoperative pain remains significant, impeding swift patient recovery. This study aimed to evaluate the differences in postoperative pain and recuperation between patients receiving intrathecal morphine paired with low-dose bupivacaine and those administered general anesthesia exclusively.

**Methods:**

This randomized controlled trial enrolled 100 patients, who were allocated into three groups: Group M (5 μg/kg morphine intrathecal injection), Group B (5 μg/kg morphine combined with bupivacaine 3 mg intrathecal injection) and Group C (intrathecal sham injection). The primary outcome was the assessment of pain relief using the Numeric Rating Scale (NRS). Additionally, intraoperative remifentanil consumption was quantified at the end of the surgery, and postoperative opioid use was determined by the number of patient-controlled analgesia (PCIA) compressions at 48 h post-surgery. Both the efficacy of the treatments and any complications were meticulously recorded.

**Results:**

Postoperative NRS scores for both rest and exercise at 6, 12, 24, and 48 h were significantly lower in groups M and B than in group C (*P*<0.05). The intraoperative remifentanil dosage was significantly greater in groups M and C than in group B (*P*<0.05), while there was no significant difference between groups M and C (*P*>0.05). There was no significant difference in intraoperative propofol dosage across all three groups (*P*>0.05). Postoperative dosages of both sufentanil and Nonsteroidal anti‐inflammatory drugs (NSAIDs) were significantly less in groups M and B compared to group C (*P*<0.05). The time of first analgesic request was later in both groups M and B than in group C (*P*<0.05). Specific and total scores were elevated at 2 days postoperative when compared to scores at 1 day for all groups (*P*<0.05). Furthermore, at 1 day and 2 days postoperatively, both specific scores and total scores were higher in groups M and B compared to group C (*P*<0.05).

**Conclusion:**

Intrathecal administration of morphine combined with bupivacaine has been shown to effectively ameliorate acute pain in patients undergoing thoracoscopic surgery.

**Trial registration:**

The trial was registered on ClinicalTrials.gov: ChiCTR2200058544, registered 10/04/2022.

## Background

Video-assisted thoracoscopic surgery (VATS) has emerged as the principal surgical intervention for lung tumors [1]. Despite its minimally invasive nature, patients frequently experience moderate to severe postoperative pain [2]. Inadequate pain management can hinder a patient’s ability to breathe deeply and expectorate sputum promptly, and may lead to secondary complications such as pneumonia and thrombosis due to decreased mobility [3]. Furthermore, it is linked to chronic post-surgical pain (CPSP), markedly impairing the patient’s recovery quality and increasing the reliance on analgesics, including opioids [4]. Acute pain involves various factors, making the anesthesiologist’s role in pain management crucial, particularly in minimally invasive procedures [5]. Techniques such as thoracic epidural analgesia (TEA) [6], paravertebral nerve blocks, anterior serratus, erector spinae, and other plane block techniques [7, 8], as well as intravenous opioid-based self-administered analgesia [9], are extensively utilized in thoracic surgeries, yet several issues persist [10–13].

The evolution of surgical techniques and the integration of robotics have substantially reduced the extent of surgical trauma, supplanting traditional open surgery with less invasive methods. A solitary intrathecal injection of small-dose morphine is straightforward to administer and capable of delivering profound analgesia at low doses, accompanied by a scant number of postoperative complications. However, the delayed onset of intrathecal morphine, with its peak effect materializing after 6 h [14], may render it inadequate for acute perioperative pain control during surgery. In contrast, bupivacaine manifests a swift onset of action. When synergistically used with morphine in the subarachnoid space, it can effectively bolster intraoperative analgesia. The use of low-dose morphine in tandem with intrathecal bupivacaine has been validated to curtail postoperative opioid use and enhance pain control during the perioperative phase in lower abdominal surgeries [15, 16]. For thoracic surgery, the analgesic potency of a solitary lumbar intrathecal morphine (ITM) injection necessitates a reassessment in light of the altered locus of surgical intervention. It stands as a potentially preferable option over high-level epidural analgesia, offering a viable alternative to TEA and PCIA [10]. This preference is due to its elevated rate of successful puncture and the diminished risks of complications such as epidural hematoma, significant dosage reduction, and a lower frequency of adverse effects [17, 18].

The objective of our research was to assess the analgesic effectiveness of intrathecal morphine in patients undergoing VATS. We specifically confirmed that the combination of intrathecal morphine and bupivacaine surpasses intrathecal morphine alone in pain relief. Additionally, we observed that this combination diminished the intraoperative consumption of remifentanil and lessened the requirement for rescue analgesics during the first 48 h post-surgery. Furthermore, we documented the complications associated with each analgesic approach.

## Methods

### Ethics and registration

This study was approved by the Medical Ethics Committee in Affiliated Hospital of Yangzhou University, Jiangsu, China (Protocol/serial number: 2022-YKL3-08–002) on 8 March 2022. All study subjects gave informed consent for all treatments and investigations. All procedures in the study involving human participants were performed in accordance with the ethics standards of the institutional and national research committee and with the Helsinki Declaration and its later amendments or comparable ethics standards. The study was conducted and reported in accordance with the Consolidating Standards of Reporting Trials (CONSORT) 2010 statement. This trial was registered at the Chinese Clinical Trial Registry (https://www.chictr.org.cn/edit.aspx?pid=164523&htm=4) on 10/4/2022 (registry number: ChiCTR2200058544).

#### Patients inclusion and exclusion criteria

Subjects to be enrolled in the study were selected prior to an elective VATS procedure.

#### Subject inclusion criteria

- An elective thoracoscopic lobectomy scheduled,
- The surgical procedure to be performed under general anaesthesia,
- Age from 18 to 64 years,
- Body Mass Index (BMI) from18 to 30 kg/m^2^,
- ASA physical status classification system class I-II.

#### Subject exclusion criteria

- Allergies to the local anesthetics, morphine, or drugs used in the study,
- Cardiac diseases, hepatic insufficiency, and renal failure,
- Subject’s refusal to participate in the study,
- Inability to understand what the study is about,
- Previous cardiothoracic surgery,
- Any contraindication to intrathecal injection (morphine allergy, coagulopathy, patient refusal),
- Patients with a history of chronic pain,
- Preoperative opioid use and/or history of opioid abuse,
- Psychiatric disease.

Patients were also excluded when failure to operate intrathecal anaesthesia, discontinuation of an intravenous analgesic pump due to a serious adverse reaction, and change of procedure.

#### Procedures

After patients were admission, oxygen was administered and ECG, HR, NBP and SPO_2_ were monitored. The right internal jugular vein was punctured and a central venous catheter was placed successfully to establish intravenous access. A radial artery puncture was performed on the healthy side for pressure measurement.

The enrolled patients were randomized by random number method into three groups: Group M (5 μg/kg morphine intrathecal injection), Group B (5 μg/kg morphine combined with bupivacaine 3 mg intrathecal injection) and Group C (intrathecal sham injection). Patients were randomly allocated 1: 1: 1 with three treatment groups using a set of computer-generated random numbers kept in sealed envelopes by an investigator not involved in clinical care. Blocks were performed before induction of general anaesthesia, and patients were not informed of their group assignments. The three groups were intrathecal injection morphine, morphine and bupivacaine, or intrathecal sham injection. The anaesthetist conducting the anaesthetic was not blinded to treatment.

Group M: Under standard monitoring, patients were placed in the left lateral position and the skin of the low back was disinfected with 0.45–0.55% povidone-iodine disinfectant centred on the L2-3 gap and sterile cavity wipes were laid. 1% lidocaine 2 ml was infiltrated locally and a type II lumbar puncture needle (5#) was used to perform an intrathecal puncture in the L2-3 space. The cerebrospinal fluid was drained freely and morphine hydrochloride 5 μg/kg was injected into the subarachnoid space within 15 s. Morphine hydrochloride configuration: dilute 1 mg morphine hydrochloride (1 mg/ml) with 0.9% sodium chloride to 10 ml (0.1 mg/ml) and inject into a 3 ml syringe after drawing at 5 μg/kg using a 1 ml syringe. Retract cerebrospinal fluid diluted to 3 ml for subarachnoid injection.

Group B: The procedure for subarachnoid puncture was the same as that for M group. 5 μg/kg of morphine hydrochloride (morphine preparation was the same as that for M group) combined with 0.75% bupivacaine 0.4 ml (3 mg) was withdrawn and diluted to 3 ml of cerebrospinal fluid for subarachnoid injection.

Group C: After the patient has cooperated in the position, the anaesthetist applies finger pressure to the skin to simulate intrathecal injection.

The 10°Trendelenburg position was used in all three groups. The flat position was resumed 20 min after completion of the operation to achieve a high plane block. Sterile dressings were given to cover the lumbar back after completion of the puncture operation in all three groups. The upper boundary of the plane of anaesthesia was measured with an alcohol swab 10–15 min after administration.

For all three groups, general anaesthesia was induced with 0.05 mg/kg midazolam, 0.4 μg/kg sufentanil and 1.5–2.0 mg/kg propofol. Cisatracurium 0.2 mg/kg was given to facilitate double-lumen bronchial tube intubation. Propofol administration was adjusted to target a Bispectral Index between 40 and 60. Cisatracurium was infused at 0.05 ~ 0.10 mg·kg^−1^·h^−1^ and remifentanil was infused at 0.1 ~ 0.25 μg·kg^−1^·min^−1^, adjusted as necessary. A single dose of azasetron (10 mg) was permitted for PONV prophylaxis. After surgery, patients were transferred to the postanaesthesia care unit for extubation.

Postoperative analgesia: All three groups received PCIA after awakening in the PACU with the same settings: sufentanil 2 μg/kg and azasetron 10 mg diluted to 100 ml with 0.9% NaCl. PCA dose, 2 ml, lockout interval 15 min. If the PCA device was fully used (NRS > 4 after three consecutive effective compressions), then aminotriol ketorolac 30 mg is administered intramuscularly for remedial analgesia. In case of postoperative nausea and vomiting, metoclopramide 10 mg is administered intramuscularly.

#### Measurement

Patients’ baseline data, surgery and anaesthesia were collected. Mean blood presssure and heart rate at eight time points were recorded: After patient admission (T0), 10 min after intrathecal block (T1), 1 min after double-lumen tracheal intubation (T2), 1 min before surgical cut (T3), 1 min after surgical cut (T4), 5 min after surgical cut (T5), at the end of surgery (T6) and 30 min after extubation (T7). IVPCA sufentanil consumption in 48 h and NRS at 1, 6, 12, 24 and 48 h postoperatively were recorded. Intra-operative consumption of remifentanil and propofol was recorded. Additionally, common side effects (sedative effect, nausea/vomiting, and pruritus) and some recovery indicators were recorded.

For sedation, the Ramsay Sedation Score was used: 1 score: irritable; 2 score: awake, quiet and cooperative; 3 score: drowsy, but able to follow commands and respond with agility; 4 score: in a state of drowsiness, can be awakened quickly; 5 score: in a state of light sleep, unresponsive to calls and responsive to stronger stimuli (e.g., tapping); and 6 score: in a state of deep sleep, not waking up to calls. If the score is greater than 4, the patient is over-sedated, and 15 μg of nalmefene is given intravenously for over-sedation. Nausea and vomiting were classified as three grades: asymptomatic, symptomatic without treatment, and symptomatic with treatment. Gastrografin 10 mg intramuscularly was given, and haloperidol 0.63–1.25 mg intravenously was given for unrelieved nausea and vomiting, which was repeated once every 8 h if necessary.

Pruritus was classified as asymptomatic, symptomatic without treatment and symptomatic with treatment, 10 mg of propofol was given for itching with the maximum dose not exceeding 50 mg. 10 mg of cetirizine tablets were given orally if itching persisted.

All patients were given a preoperative urinary catheter, which was removed on the second day. If urinary retention occurred after removal of the urinary catheter, hot compresses and massage were used, and the catheter was reintroduced if it could not be relieved.

All results were recorded by an anaesthetist who had no knowledge of the subgroups. The scores of Quality of Recovery-15 (QoR-15) is the recently developed to validate short-form postoperative recovery evaluation. QoR-15 questionnaire has 15 questions that assess patient-reported postoperative health status using a 11-point numerical rating scale that leads to a minimum score of 0 (poor recovery) and a maximum score of 150 (excellent recovery). QoR-15 was obtained 24 h and 48 h after surgery in this trail.

The primary outcome was the pain scores at rest and with cough at 48 h after surgery. Secondary outcomes were intraoperative propofol and remifentanil consumption, intraoperative haemodynamic variables, postoperative rescue analgesic requirements and adverse events; and postoperative recovery indicators.

#### Statistical analysis

Variables were summarized as mean ± SD for normally distributed continuous variables (including demographic profile of the patients, duration of surgery, fluid intake and output, hemodynamic changes, Intra-operative remifentanil use, propofol use, first feeding time, first time out of bed, QoR-15 score), and intergroup comparisons were performed using one-way analysis of variance (ANOVA). Median (25th to 75th percentile) for continuous variables with evidence of nonnormality or for ordinal variables (Hospital stay days, blood loss, pain scores, cumulative amount of IVPCA sufentanil used, time of first analgesic request, post-operative extubation time), comparisons between groups were made using the Kruskal–Wallis test. Frequency counts and percentages for categorical variables were analysed using the Pearson’s chi-squared test (sex, ASA physical status, underlying disease, chest drain days, nausea/vomiting) or Fisher’s exact test (pruritus). Statistical analysis was performed using statistical software SPSS (25.0; IBM Corp., Armonk, NY, USA). The 95% confidence intervals of the median differences were estimated by bootstrap method based on resampling through Python version 2.7.6. The significance level was set at *P* < 0.05.

#### Sample size considerations

48 h post-operative coughing on the NRS scores was defined as a clinically meaningful effect. Assuming a standard deviation of 2 for each of the three pain score outcomes on the basis of a preliminary study. The pretest result was an NRS score of 1.3 for group M, 1.2 for group B and 3.3 for group C at 48 h postoperative coughing. A sample size of 20 patients per group had 90% power to detect effectiveness of intrathecal morphine and intravenous analgesic pumps on all three primary outcomes at the 0.05 significance level. To allow for some individuals not completing the trial, 33 patients were recruited in each treatment group.

## Results

**Fig. 1:**
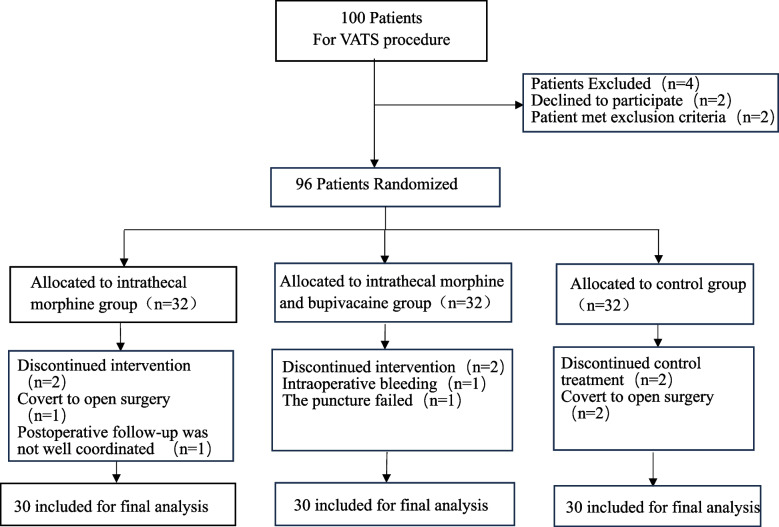
Consolidated standards of reporting trials diagram.VATS,Video-assisted thoracoscopic surgery

A total of 100 patients were randomised from 15 March 2022 to the 25 December 2022. Ten patients were excluded for various reasons, leaving 30 patients given general anaesthesia alone, 30 patients given intrathecal morphine and 30 patients given intrathecal morphine and bupivacaine (the study flow diagram is displayed in Fig. 1). The demographic profile of the patients,including sex, age, BMI, ASA classification, exhibited no significant differences in three groups. Underlying diseases, Chest drain, duration of surgery, one lung ventilation time and blood loss also did not differ significantly between the groups (*P*>0.05), as illustrated in Table 1.

**Table 1:** Baseline characteristics of the study groups

	Group M (*n*=30)	Group B (*n*=30)	Group C (*n*=30)	*P*
Gender, N (%): Female	17	20	18	0.721
Gender, N (%): Male	13	10	12	0.721
Age (years)	56.4±4.6	55.7±5.7	57.8±4.6	0.281
Weight (kg)	68.2±8.9	66.4±7.7	65.5±9.3	0.471
Height (cm)	167.8 ± 7.4	166.8 ± 6.2	166.9 ± 7.0	0.825
BMI (kg/m^2^)	24.2 ± 2.8	23.9 ± 3.0	23.5 ± 3.0	0.639
ASA physical status (1/2)	14/16	16/14	15/15	0.875
Hb(g/L)	132.2 ± 5.1	131.9 ± 5.0	133.1 ± 5.5	0.642
WBC (cells/mm^3^)	6.44 ± 1.71	6.57 ± 2.00	6.06 ± 1.80	0.545
Platelet (cells/mm^3^)	167.4 ± 30.1	172.3 ± 34.5	180.3 ± 44.0	0.390
Albumin (unit)	40.0 ± 1.3	40.1 ± 1.4	39.9 ± 1.4	0.800
Underlying disease(s)				
Diabetes mellitus	4 (13.3%)	5 (16.7%)	7 (23.3%)	0.587
Hypertension	10 (33.3%)	11 (36.7%)	12 (40%)	0.866
Dyslipidemia	9 (30%)	6 (20%)	7 (23.3%)	0.656
Smoking	9 (30%)	6 (20%)	7 (23.3%)	0.656
Chest drain(3/4/5 days)	6/16/8	5/13/12	5/15/10	0.839
Duration of surgery(min)	86.8 ± 17.5	84.8 ± 16.7	88.3 ± 20.9	0.763
One lung ventilation time(min)	74.3 ± 18.3	72.5 ± 17.4	70.5 ± 21.1	0.737
Hospital stay(days)	5 (4, 6)	5 (4, 6)	5 (4, 5)	0.192
Fluid (ml)	821.7 ± 147.2	860.0 ± 141.1	900.0 ± 177.1	0.157
Blood loss (ml)	75 (60, 90)	80 (57.5, 90)	75 (50, 82.5)	0.640
Urine output (ml)	190.0 ± 38.7	184.0 ± 36.6	180.0 ± 55.1	0.698
Reoperation	0	0	0	-

*Abbreviations*: *ASA* American Society of Anesthesiologists; *BMI* Body mass index; *Hb* Hemoglobin, *WBC* White blood cell

Blood pressure in the three groups showed an overall decreasing trend from T0-T3, and gradually increased and stabilised from T4 onwards due to surgical stimulation. Overall, the change in MAP at the moment of T0-T7 in the three groups of patients tends to stabilise and is around 90, with no significant difference in comparison (*P*>0.05). The HR of the three groups of patients tended to be stable at the moment of T0-T7, all fluctuating above and below 70, and there was no significant difference in comparison (*P*>0.05), as presented in Table 2.

**Table 2:** Hemodynamic changes of the study groups at different moments

	Group M (*n*=30)	Group B (*n*=30)	Group C (*n*=30)	*P*-value^a^
Blood pressure				
T0	94.5 ± 9.9	95.6 ± 11.6	93.4 ± 12.3	0.751
T1	92.1 ± 8.0	93.6 ± 10.1	94.6 ± 10.7	0.607
T2	92.9 ± 9.1	95.9 ± 9.7	96.7 ± 12.8	0.344
T3	91.1 ± 8.4	90.7 ± 7.6	87.3 ± 9.8	0.181
T4	91.7 ± 10.0	95.4 ± 8.7	94.2 ± 11.0	0.349
T5	93.9 ± 9.2	97.4 ± 10.3	93.6 ± 10.3	0.264
T6	93.5 ± 6.4	93.8 ± 8.2	96.4 ± 8.2	0.277
T7	94.4 ± 5.4	93.2 ± 6.4	95.1 ± 5.8	0.438
Heart rate				
T0	74.7 ± 7.7	76.6 ± 9.7	75.1 ± 8.4	0.682
T1	75.9 ± 8.2	74.3 ± 9.5	77.0 ± 10.1	0.513
T2	74.0 ± 9.5	74.5 ± 12.2	76.4 ± 10.3	0.672
T3	73.5 ± 8.4	69.6 ± 7.1	73.1 ± 7.6	0.101
T4	73.5 ± 8.5	69.3 ± 9.0	71.9 ± 9.0	0.183
T5	72.6 ± 6.3	69.3 ± 6.7	71.8 ± 7.5	0.166
T6	71.2 ± 7.4	66.5 ± 9.4	70.3 ± 7.1	0.058
T7	71.7 ± 6.0	69.6 ± 6.8	72.2 ± 8.5	0.343

*Abbreviations*: *T0* After patient admission, *T1* 10 min after intrathecal block, *T2* 1 min after double-lumen tracheal intubation, *T3* 1 min before surgical cut, *T4* 1 min after surgical cut, *T5* 5 min after surgical cut, *T6* at the end of surgery and *T7* 30 min after extubation

In all three groups of pain scores, the scores increased and stabilised over time. The rest and exercise NRS scores at 6 h, 12 h, 24 h and 48 h postoperatively were significantly lower in groups M and B compared to group C (*P*<0.05). There was no significant difference in rest and exercise NRS scores at 6 h, 12 h, 24 h and 48 h postoperatively in groups M and B. There was no statistically significant difference in resting and motor NRS scores at 1 h postoperatively between the three groups (*P*>0.05). Intraoperative remifentanil dosage was significantly higher in groups M and C (0.77 ± 0.20, 0.87 ± 0.27) compared to group B (0.52 ± 0.14) (*P*<0.05). The difference in intraoperative remifentanil dosage between groups M and C was not statistically significant (*P*>0.05). The difference in intraoperative propofol dosage(429.2 ± 104.0, 411.9 ± 91.0, 377.0 ± 103.5) between the three groups was not statistically significant (*P*>0.05). Postoperative sufentanil and NSAID dosage was significantly reduced in groups M and B compared to group C (*P*<0.05). The time of first analgesic request was later in both groups M and B than in group C (*P*<0.05). There was no statistically significant difference in group M compared to group B(*P*>0.05), as presented in Table 3.

**Table 3:** Post-operative outcomes (pain scores)

Variables	Group M (*n*=30)	Group B (*n*=30)	Group C (*n*=30)	*P*-value^a^
Pain at rest				
At 1 h	0 (0 ~ 1.0)	0 (0 ~ 1.0)	0.5 (0 ~ 1.0)	0.509
At 6 h	0.5 (0 ~ 1.0)	0 (0 ~ 1.0)	2.0 (2.0 ~ 2.3)	0.000
At 12 h	1.0 (0 ~ 1.0)	1.0 (0 ~ 1.0)	3.0 (2.0 ~ 3.0)	0.000
At 24 h	1.0 (0 ~ 2.0)	1.0 (0 ~ 2.0)	2.0 (2.0 ~ 3.0)	0.000
At 48 h	1.0 (0 ~ 1.0)	1.0 (0 ~ 1.0)	2.0 (2.0 ~ 3.0)	0.000
Pain with cough				
At 1 h	1.0 (1.2 ~ 2.0)	1.0 (1.0 ~ 2.0)	2.0 (1.0 ~ 2.0)	0.071
At 6 h	2.0 (1.0 ~ 2.0)	1.0 (1.0 ~ 2.0)	3.0 (3.0 ~ 4.0)	0.000
At 12 h	2.0 (2.0 ~ 3.0)	2.0 (1.0 ~ 3.0)	4.0 (3.0 ~ 4.0)	0.000
At 24 h	2.0 (1.8 ~ 3.0)	2.0 (2.0 ~ 3.0)	4.0 (3.0 ~ 4.0)	0.000
At 48 h	2.0 (1.0 ~ 3.0)	2.0 (2.0 ~ 2.3)	3.5 (3.0 ~ 4.0)	0.000
Intra-operative remifentanil use(mg)	0.77 ± 0.20	0.52 ± 0.14	0.87 ± 0.27	0.000
Propofol use(mg)	429.2 ± 104.0	411.9 ± 91.0	377.0 ± 103.5	0.125
Cumulative amount of IVPCA sufentanil used in 48 h (mg)	0 (0,2.8)	0 (0,0.6)	5.2 (2.8,7.6)	0.000
Time of first analgesic request	14.5 (10.5,17.5)	18(14,20)	8(6,10)	0.000
Additional non-opioid analgesia	0 (0,0)	0(0,0)	30 (0,60)	0.000

*Abbreviations*: *IVPCA* Intravenous patient-controlled analgesia, *ITM* Intrathecal morphine

Time of first analgesic request:

Group 1—Group 2 95%CI: [-6.0, -3.0], Group 1—Group 3 95%CI: [-11.0, -6.0], Group 2—Group 3 95%CI: [-8.0, -3.0]

No over-sedation and urinary retention occurred in any of the three groups within 48 h. Pruritus occurred in 3 cases (10%) in group M and 2 cases (6.6%) in group B and resolved on its own without medication (*P*>0.05). There was no significant difference in the incidence of nausea and vomiting (36.7%, 33.3% and 30%, respectively) among the three groups, and those who experienced nausea and vomiting were mostly mild and symptomatic but did not require treatment(*P*>0.05), as illustrated in Table 4.

**Table 4:** Common opioid-related side effects (sedative effect, nausea/vomiting, and pruritus)

Variables	Group M (*n*=30)	Group B (*n*=30)	Group C (*n*=30)	*P*-value^a^
**Sedative effect**				
Having sedative event within 48 h, n (%)	0	0	0	-
**Pruritus**				
Having pruritus within 48 h post-operation, n (%)	3 (10)	2 (6.6)	0	0.227
No symptom	0	0	0	**-**
Having symptom without treatment	3	2	0	0.227
Having symptom with treatment	0	0	0	**-**
**Nausea/Vomiting**				
Having nausea/vomiting within 48 h post-operation, n (%)	11 (36.7)	10 (33.3)	9 (30)	0.861
No symptom	1 (9.1)	1 (10)	1 (11.1)	0.840
Having symptom without treatment	7(63.6)	4 (40)	4 (44.4)	0.840
Having symptom with treatment	3 (27.3)	5 (50)	4 (44.4)	0.840
**Urinary retention**				
48 h incidence	0	0	0	-
Re-insertion of urinary catheter	0	0	0	-

^a^Chi-squared test or Fisher’s exact tests

The postoperative extubation time of the three groups of patients was close to about 20 min, and no significant difference between the three groups(*P*>0.05). The time of first feeding in three groups (8.8 ± 1.2,8.6 ± 1.1,8.6 ± 1.3) and the time of first getting out of bed (19.6 ± 1.8,19.3 ± 1.6,19.1 ± 1.6), and the differences were no significant effect(*P*>0.05), as presented in Table 5.

**Table 5:** Post-operative recovery indicators($\overline{x }$±s)

Variables	Group M (*n*=30)	Group B (*n*=30)	Group C (*n*=30)	*P*
Post-operative extubation time (min)	20(19.5, 30)	20(17.3, 25)	20(15, 30)	0.793
First feeding time(h)	8.8 ± 1.2	8.6 ± 1.1	8.6 ± 1.3	0.649
First time out of bed (h)	19.6 ± 1.8	19.3 ± 1.6	19.1 ± 1.6	0.467

In all three groups, the specific scores including pain, physical comfort, physical independence, psychological support, emotional support were higher at 2d postoperatively compared to 1d postoperatively (*P*<0.05); the total scores were higher at 2d postoperatively compared to 1d postoperatively(123.0 ± 5.9, 125.7 ± 7.3, 112.1 ± 4.4 vs 110.0 ± 4.8, 112.6 ± 6.0, 96.9 ± 4.7, *P*<0.05). At 1d and 2d postoperatively, five-part scoring were higher in the M and B groups compared to the C group (*P*<0.05); the total scores were higher in the M and B groups compared to the C group (*P*<0.05); there was no significant difference between groups M and B when comparing them in each of the scores and in the total score(110.0 ± 4.8 vs 112.6 ± 6.0, 123.0 ± 5.9 vs 125.7 ± 7.3), as shown in Table 6.

**Table 6:** Post-operative QoR-15 score ($\overline{x }$±s)

	POD1			*P* value	POD2			*P* value
	Group M (*n*=30)	Group B (*n*=30)	Group C (*n*=30)		Group M (*n*=30)	Group B (*n*=30)	Group C (*n*=30)	
Domain ‘pain’	15.0 ± 1.3	15.6 ± 1.4	12.6 ± 1.4	0.000	17.3 ± 1.7	17.5 ± 1.9	15.2 ± 1.3	0.000
Domain ‘physical comfort’	35.9 ± 2.6	37.0 ± 2.9	30.6 ± 2.7	0.000	41.1 ± 2.9	42.5 ± 3.1	37.4 ± 2.4	0.000
Domain ‘physical independence’	14.4 ± 1.3	14.7 ± 1.5	13.4 ± 1.9	0.006	16.0 ± 1.3	16.4 ± 1.5	15.1 ± 1.9	0.006
Domain ‘psychological support’	16.1 ± 0.9	16.1 ± 1.3	13.7 ± 1.3	0.000	16.9 ± 1.3	16.9 ± 1.2	14.3 ± 1.2	0.000
Domain ‘emotional support’	28.6 ± 2.6	29.2 ± 2.5	26.7 ± 1.9	0.000	31.6 ± 2.6	32.5 ± 2.8	30.0 ± 2.1	0.001
QoR-15	110.0 ± 4.8	112.6 ± 6.0	96.9 ± 4.7	0.000	123.0 ± 5.9	125.7 ± 7.3	112.1 ± 4.4	0.000

## Discussion

This prospective, interventional, single-blind, randomized study reveals that the combination of intrathecal morphine and bupivacaine offers effective analgesia for patients undergoing VATS. Compared to conventional general anesthesia, this anesthesia approach delivered superior pain control during the first 48 h post-surgery, leading to a lower cumulative consumption of IVPCA sufentanil and a reduced necessity for rescue analgesics over the same period. Compared to conventional general anesthesia, this anesthesia approach delivered superior pain control during the first 48 h post-surgery, leading to a lower cumulative consumption of IVPCA sufentanil and a reduced necessity for rescue analgesics over the same period. Aligning with prior research, our findings suggest that intrathecal morphine and bupivacaine are viable alternatives to epidural blocks, particularly for thoracoscopic procedures that necessitate higher levels of analgesia. The expedited administration process of morphine and bupivacaine intrathecal injections, as opposed to the longer duration required for intrathecal morphine alone, potentially renders them more suitable for clinical use. Our results corroborate that a singular intrathecal injection of low-dose morphine in conjunction with bupivacaine can effectively bridge the gap in analgesia due to the gradual onset of intrathecal morphine’s effects [19].

Intrathecal morphine, when used alongside sufentanil and combined with an epidural infusion of bupivacaine and fentanyl in the thoracic segment, has been demonstrated to provide analgesic effects equivalent to those achieved in post-thoracoscopic analgesia [20]. Moreover, intrathecal morphine has been reported to deliver satisfactory postoperative analgesia following total endoscopic coronary artery bypass grafting [21]. Our results further substantiate the exceptional analgesic performance of ITM in VATS procedures. As delineated in Table 3, intrathecal morphine administration was associated with approximately a 20% reduction in intraoperative remifentanil usage, whereas the combined use of morphine and bupivacaine via intrathecal injection achieved around a 40% reduction. Under the guidance of BIS monitoring, the propofol dosage was consistent across all groups. Given the gradual onset of ITM, bupivacaine is often employed to mitigate this delay. Based on the findings by Motamed et al. [22], our study utilized 3 mg of bupivacaine to circumvent the adverse effects linked with higher doses. Low doses of bupivacaine have the advantage of selectively obstructing nociceptive and temperature sensations, while only causing a mild sympathetic and motor nerve blockade, which does not lead to significant hemodynamic changes. In Table 2, the comparison of MAP and HR across the three groups did not reveal any statistical differences at any of the measured intervals. Echoing the work of Ban et al. [23], the employment of 5 mg of bupivacaine in conjunction with ITM for hepatectomy was also shown to maintain stable hemodynamic parameters (HR, MBP).

In our study, we utilized the 10 Trendelenburg position, ensuring rapid intrathecal drug administration within 15 s to aid the dispersion of the agents to the thoracic region. The sensory block level was assessed with an alcohol swab, achieving a blockade up to the T2 dermatome prior to anesthesia induction. This effectively corresponded with the T2 to T10 dermatomal pain regions implicated in VATS incisions, providing effective analgesia during the operation and minimizing the necessity for the intraoperative opioid remifentanil.

The study by Dhawan et al. [21] highlighted that a lower dose of ITM at 5 μg/kg was both safe and effective for postoperative analgesia in total endoscopic coronary artery bypass grafting. This dose is significantly less than what has been traditionally used for postoperative analgesia [24], suggesting that effective pain management can be achieved with reduced doses. Importantly, their findings indicated that this reduced dose did not lead to postoperative respiratory depression, a serious concern often associated with opioid administration. Preoperative catheterization was employed in this study to prevent urinary retention, a common complication with ITM. Successful removal of urinary catheters on the second day post-procedure signifies a satisfactory resolution of this potential side effect. Nausea and vomiting are frequently encountered complications following thoracic surgery, with occurrence rates ranging from 25 to 60% [25]. Despite prophylactic administration of azelastine, as delineated in Table 4, the prevalence of nausea and vomiting persisted at 30%, exhibiting no significant variance across the three studied groups (*P* > 0.05). Consequently, it becomes evident that the current approach to managing nausea and vomiting in perioperative analgesia necessitates enhancement, advocating for a diversified pharmacological strategy to preemptively mitigate these effects. Furthermore, pruritus ranks as a predominant adverse reaction to ITM. As reported in Table 4, a subset of 5 patients in the two ITM cohorts experienced mild pruritus, localized primarily to the upper body. This condition did not compromise patient well-being nor necessitate specialized intervention. Notably, the incidence was inferior to that reported in Dhawan’s study, at 21.2% [21]. This suggests that the utilization of propofol and azastazuron in this investigation may have attenuated the pruritic effects associated with ITM within the two groups [26].

As the ERAS paradigm advances, the adoption of accelerated-track anesthesia is increasingly recommended. This strategy emphasizes prompt postoperative awakening, expeditious extubation, and early mobilization. Referencing Table 5, none of the patients in either the M group or the B group experienced delayed recovery or extubation, aligning with findings from Suksompong et al. [27]. In stark contrast, the ITM group observed in Chaney et al.’s [28] clinical trial faced extubation delays, possibly attributable to the administration of a higher initial dose of ITM. This serves as a cautionary note on the necessity of rigorous dosage regulation when employing ITM, underscoring the importance of determining and administering the minimal effective dose.

The Quality of QoR-40 is recognized as a significant measure of patient health status during the initial postoperative phase [29], yet its practical application has been criticized for being protracted and complex. The more recent Quality of QoR-15 score has gained international consensus as a crucial metric in clinical trials to evaluate postoperative patient comfort enhancements [30]. According to the findings presented in Table 6, patients in both the M and B groups exhibited superior physical comfort, enhanced emotional wellbeing, and greater overall QoR-15 scores on the first and second day following surgery. In contrast, those receiving only postoperative PCIA experienced suboptimal pain control, decreased comfort levels, an increased occurrence of dysphoria, and an overall diminished quality of recovery.

This study is not without its limitations. Foremost, the relatively modest sample size and the exclusion of high-risk patients may introduce a degree of bias into the findings. Additionally, the investigation was confined to a single dose of ITM at 5 μg/kg, precluding a comparative analysis across a spectrum of dosages.The experiments of this group are continuing and will further explore the appropriate dose to apply to the patient. Finally, the scope of this research was limited to the assessment of early postoperative recovery quality, neglecting the potential effects on long-term recuperation and the development of chronic pain in patients. For the long-term effects, the results of studies using multi-centre and large samples may be more convincing, and our group is also trying to collaborate with a number of hospitals to conduct multi-centre studies, and expects to demonstrate satisfactory results in the future.

## Conclusions

Intrathecal injection of 5 μg/kg morphine (with or without 3 mg bupivacaine) in patients undergoing thoracoscopic lobectomy significantly improved acute postoperative pain, reduced postoperative analgesic dose, improved quality of postoperative recovery and increased patient satisfaction compared to the application of postoperative PCIA. 5 μg/kg morphine combined with 3 mg bupivacaine intrathecally reduced intraoperative opioid doses without causing hemodynamic fluctuations.

## Data Availability

The datasets analyzed during the current study are available from the corresponding author upon reasonable request.
